# Self-Assembly System Based on Cyclodextrin for Targeted Delivery of Cannabidiol

**DOI:** 10.3389/fchem.2021.754832

**Published:** 2021-11-08

**Authors:** Panyong Zhu, Pin Lv, Yazhou Zhang, Rongqiang Liao, Jing Liu, Rong Guo, Xuan Chen, Xiali Liao, Chuanzhu Gao, Kun Zhang, Ming Yang, Bo Yang

**Affiliations:** ^1^ Faculty of Life Science and Technology, Kunming University of Science and Technology, Kunming, China; ^2^ Industrial Crop Research Institute, Yunnan Academy of Agricultural Sciences, Kunming, China; ^3^ Pharmacy Department, Chongqing Emergency Medical Center, Chongqing University Central Hospital, Chongqing, China; ^4^ The Affiliated of Stomatology, Kunming Medical University, Kunming, China; ^5^ School of Agriculture, Yunnan University, Kunming, China

**Keywords:** biotin, ß-cyclodextrin, cannabidiol, inclusion complex, targeting delivery, self-assembly

## Abstract

Cannabidiol (CBD) is one specific kind of the cannabinoid in *Cannabis sativa L* with a wide range of pharmacological activities. However, the poor water solubility and specificity of CBD limits its application in pharmaceutical field. For solving these problems, in this work, we successfully prepared a targeted carrier by grafting biotin (BIO) onto ethylenediamine-β-Cyclodextrin (EN-CD) in a single step to generate a functionalized supramolecule, named BIO-CD. Subsequently, an amantadine-conjugated cannabinoids (AD-CBD) was prepared and self-assembled with the BIO-CD. A series of methods were used to characterize the inclusion behavior and physicochemical properties of AD-CBD and BIO-CD. The results showed that AD-CBD entered the cavity of BIO-CD and formed a 1:1 host-guest inclusion complex. MTT assay and confocal laser scanning microscopy (CLSM) revealed that the targeting effect and anticancer activity of AD-CBD/BIO-CD inclusion complex against three human cancer cell lines were higher than BIO-CD, AD-CBD and free CBD. Moreover, the inclusion complex could release drugs under weakly acidic conditions. These results demonstrated that AD-CBD/BIO-CD inclusion complex possess excellent targeted and anticancer activity, which is hopeful to be applied in clinic as a new therapeutic approach.

## Introduction

Cannabaceae, an annual herbaceous crop, has been widely cultivated for fiber, food, and medicine for thousands of years (Pain, 2015; Guo et al., 2018). Hemp [cultivated *Cannabis* with lower Δ^9^-tetrahydrocannabinol (THC)] has received increasing attention in recent years, especially for cannabinoids (CBD) (Lv et al., 2019; Kotschenreuther et al., 2020). CBD is a component of cannabis with no psychoactive properties (Vrechi et al., 2021). Using an inverse agonist of CB2 receptors, an agonist of TRPA1, TRPV1, TRPV2, 5HT-1A, and PPARγ receptors, as well as an antagonist of CB1 receptor and GPR55 (Pisanti et al., 2017; Rong et al., 2017; Ferro et al., 2018; Raymundi et al., 2020). CBD also exhibits a wide range of anti-tumor activities, which can induce programmed death of different types of cancer cells according to reports (Olea-Herrero et al., 2009; Aviello et al., 2012; Pacher, 2013; Fonseca et al., 2018; Yang et al., 2020), including human prostate cancer, human breast carcinoma, human glioblastoma, human cervical cancer, human lung cancer, lymphocytic, and monocytic leukemias, endometrial cancer, pancreatic cancer. However, the poor water solubility and specificity of CBD severely limits its application in the medical field (Li et al., 2021). Therefore, it is of great significance to improve the solubility and specificity of CBD.

How to build an advanced drug delivery system, while making it has high bioavailability, fewer side effects and the best therapeutic effect remains challenging (Liao et al., 2020; Bartusik-Aebisher et al., 2021). Small molecule hydrophobic drugs as guest can from the host-guest inclusion complexes with macrocycles as host to effectively improve the physical and chemical properties of those drugs (Zhou et al., 2017; Kaur et al., 2019). Cyclodextrins is one of the safe and effective excipients in pharmaceutical preparations, which can improve the solubility, absorption, and bioavailability of hydrophobic drugs (Del Valle, 2004).

The development of molecular biology and cell biology has promoted the emergence of targeted drugs for the treatment of cancer. (Pang et al., 2019; Hajeri et al., 2020). Targeting technology facilitates drug delivery because it deposits the drug in the desired location to achieve the most effective treatment of the disease (Zhang et al., 2018a). An obvious solution to the lack of targeting ability of natural cyclodextrin is to modify the targeting group of cyclodextrin. Biotin (BIO), also known as vitamin H, is a good targeting ligand, as its receptor can be overexpressed in many cancer cells (Singh et al., 2010; Rompicharla et al., 2019; Zhang et al., 2018b) such as lung cancer cells, colon cancer cells, kidney cells, ovarian cancer cells, and breast cancer cells. More excitingly, it is rarely expressed in normal cells (Lv et al., 2017). Furthermore, when the functional groups are exposed outside the cavity of CDs, the anti-tumor activity of most drugs can be enhanced (Jiao et al., 2012; Zhang et al., 2018a). For this reason, amantadine was used to modify CBD (AD-CBD). Adamantane interacts with cyclodextrin to form a stable complex, ultimately exposing the CBD to the outside of the CDs cavity (Rompicharla et al., 2018). The characterization and inclusion behavior of inclusion complex of amantadine conjugated cannabinoid (AD-CBD) and biotin-ethylenediamine-β-cyclodextrin (BIO-CD) in solution and solid state were studied by various methods, while the water solubility and anti-tumor activity of the inclusion complex were tested in our study.

## Experimental

### Materials

Biotin (MW = 244.3, 98% purity) and ß-cyclodextrin (MW = 1,135.0, 98% purity) were purchased from Adamas Reagent (Shanghai, China). Cannabidiol (MW = 314.2, purity > 98%) was collected from Yunnan Academy of Agricultural Sciences (Kunming, China), and they can be used without further purification. All chemicals and experiments were carried out by analytical grade and ultrapure water.

### Methods

#### Synthesis of AD-CBD

The CBD (628 mg, 2 mmol) was dissolved in 10 ml DMF (N,N-Dimethylformamide), and then DMAP (4-dimethylaminopyridine) (24 mg, 4.5 mmol) and succinic anhydride (334 mg, 2.1 mmol) were added in sequence at room temperature. After stirring at room temperature for 30 min, the reaction was detected by TLC. The reaction solution was diluted with ethyl acetate, extracted with saturated NH_4_Cl solution and water. Then the organic layer was dried with anhydrous Na_2_SO_4_, and purified by column chromatography to obtain the product CBD-COOH (534 mg, yield: 64.5%).

CBD-COOH (414 mg, 1.0 mmol) was dissolved in DMF of 7 ml EDCI (1-ethyl-3 (3-dimethylpropylamine) carbodiimide) (277.5 mg, 1.5 mmol) and NHS (N-hydroxysuccinimide) (172.5 mg, 1.5 mmol) were added successively into the solution in ice bath. The solution was stirred for 30 min under the same condition. And AD (Amantadine) (188 mg, 1 mmol) and TEA (Trimethylamine) (150 μl, 1 mmol) were added into the mixture at room temperature. The reaction was stirred at 50°C for 24 h. Finally, the solution was dropped into 100 ml cold water, resulting in white precipitate. The white solid amantadine-cannabidiol (AD-CBD) was obtained by column chromatography purified and vacuum freeze-drying (453 mg, yield: 82.8%).

#### Preparation of Solid Inclusion Complex of AD-CBD/BIO-CD

The AD-CBD/BIO-CD solid inclusion complex was prepared by the method of suspension. AD-CBD and BIO-CD (molar ratio 3:1) were added to water, and the resulting suspension was stirred at 25°C in dark conditions for 72 h. Then it was filtered through a 0.45 μm aperture microporous membrane to remove any undissolved solids. Finally, the filtrate was lyophilized in vacuum to produce a solid inclusion complex of AD-CBD/BIO-CD.

#### Preparation of Physical Mixtures of AD-CBD and BIO-CD

At room temperature, the AD-CBD and BIO-CD with a molar ratio of 1:1 were thoroughly mixed in a small mortar to obtain a physical mixture.

#### Scanning Electron Microscope Analysis

The microscopic morphology of solid inclusion complex in solid state was detected with the help of scanning electron microscope (TESCAN, model VEGA3). The solid inclusion complex of AD-CBD/BIO-CD, along with AD-CBD, BIO-CD and their physical mixtures were dispersed on the double-sided adhesive tape, it was fixed on a brass stub before the test. Gold powders were sprayed on the samples to make them electrically conductive. Then, under reduced pressure conditions, the micrographs were obtained by using 30 kV acceleration potential.

#### Fourier Transform Infrared Spectroscopy Spectra

The absorbent data of the AD-CBD, BID-CD and their solid inclusion complex and physical mixtures were evaluated on Bruker infrared spectrometer (VERTEX 80v) in KBr.

#### Powder X-Ray Diffraction

D/Max-3B diffractometer (Japan, Cu-Kα, λ = 1.5460 Å) was used for the solid inclusion complex of AD-CBD/BIO-CD, as well as AD-CBD, BIO-CD, and physical mixtures to obtain their XRD patterns under the following conditions: Voltage: 40 kV; Scanning speed: 5°min^−1^; Scan between 5° and 60° in steps of 2θ = 0.02°.

#### Thermal Analysis

Thermogravimetric analysis (TG) and differential scanning calorimetry (DSC) thermograms of AD-CBD, BIO-CD, and their physical mixture and solid inclusion complex were recorded on a NETZSCH STA449F3 instrument (Germany) with a 10°Cmin^−1^ heating rate from 25°C (room temperature) to 500°C under a N_2_ flow of 100 ml min^−1^.

#### Standard Curve of AD-CBD

The AD-CBD solutions (KH_2_PO_4_/K_2_HPO_4_ buffer solution, pH 7.0) were configured with a series of concentration ranges: 0.15–0.4 mM (0.15, 0.20, 0.25, 0.30, 0.35, and 0.40 mM). Under the condition of 25°C, the absorbance was recorded at 280 nm with UV-2550 to obtain a fitted standard curve. The concentration (C, mM) was the x-coordinate and the absorbance was y-coordinate (Supplementary Figure S1). It was concluded that the standard curve of AD-CBD could be expressed by the equation: A = 1.4904C + 0.0174 (*R* ^2^ = 0.9992).

#### Job Plot Analysis

The Job plot is a continuous variation technology, and used to determine the stoichiometry of inclusion typically. Owing to the poor water solubility of AD-CBD, we use methanol with similar physicochemical properties to complete this experiment, so as to reduce the difference with biological system as much as possible. We tried to simulate the biological systems with buffer solutions with different pH values to do this experiment, but did not get the results we want. After consulting the literature, we decided to do the Job’s plot experiment use the 1:1 methanol: water solvent system (Li et al., 2021). The equimolar (1 × 10^–3^ M) AD-CBD and BIO-CD mixed into a constant volume (1 ml: 9 ml; 2 ml: 8 ml; 3 ml: 7 ml, etc.) in methanol-water solutions (55:45, v/v). After stirring at 25°C for 2 h, the sample was filtered by a 0.45 μm aperture microporous membrane. UV-Vis spectroscopy (Shimadzu UV-2550) was used to measure the absorbance (Abs) of each solution at 280 nm, and the difference between the Abs with and without BIO-CD were determined by ΔAbs. Then, the Job plot was obtained by drawing ΔAbs × F and F (calculated by Eq. 1).

$$\text{F}=\frac{\left[\text{AD}-\text{CBD}\right]}{\left[\text{AD}-\text{CBD}\right]+\left[\text{BIO}-\text{CD}\right]}$$ (1)

#### ^1^H Nuclear Magnetic Resonance Titration

In this section, the binding constants of BIO-CD and AD-CBD were studied by using ^1^H NMR titration. As the solubility of AD-CBD in water was very poor, it was necessary to use 50 mM DMSO-d_6_ in D_2_O when performing ^1^H NMR titration, so that AD-CBD had sufficient solubility (about 1 mM) for detection by NMR. In this experiment, the concentration of one component was unchanged, while changed the concentration of the second component. A series of samples were designed. Similarly, ^1^H NMR titration was used to determine the binding constant (Ks) of these host-guest systems, and the titration curve combined with the nonlinear least-square was fitted based on the data and the 1:1 model. (Multiple articles had confirmed that adamantane forms a 1:1 inclusion complex with ß-cyclodextrin). The chemical shift of H (AD) and binding constant (Ks) were described as follows:

H+G=C (2)

$$\delta =\delta_{\text{H}}\left(1-\frac{\left[\text{C}\right]}{{\left[\text{H}\right]}_{\text{t}}}\right)+\delta_{\text{C}\frac{\left[\text{C}\right]}{{\left[\text{H}\right]}_{\text{t}}}}$$ (3)

$${\left[\text{H}\right]}_{\text{t}}\left(\delta -\delta_{\text{H}}\right)=\left[\text{C}\right]\left(\delta_{\text{C}}-\delta_{\text{H}}\right)$$ (4)

$${\text{K}}_{\text{s}}=\frac{\left[\text{C}\right]}{\left[\text{H}\right]+\left[\text{G}\right]}$$ (5)

$${\left[\text{H}\right]}_{\text{t}}=\left[\text{H}\right]+\left[\text{C}\right]$$ (6)

$${\left[\text{G}\right]}_{\text{t}}=\left[\text{G}\right]+\left[\text{C}\right]$$ (7)

$${\text{K}}_{\text{s}}=\frac{\left[\text{C}\right]}{\left({\left[\text{H}\right]}_{\text{t}}-\left[\text{C}\right]\right)\left({\left[\text{G}\right]}_{\text{t}}-\left[\text{C}\right]\right)}$$ (8)

$$\left[\text{C}\right]=\frac{\left({\left[\text{H}\right]}_{\text{t}}+{\left[\text{G}\right]}_{\text{t}}+1/{\text{K}}_{\text{a}}\right)\pm \sqrt{{\left({\left[\text{H}\right]}_{\text{t}}+{\left[\text{G}\right]}_{\text{t}}+1/{\text{K}}_{\text{s}}\right)}^{2}-4\left[{\text{H}}_{\text{t}}\right]{\left[\text{G}\right]}_{\text{t}}}}{2}$$ (9)

$$\delta -\delta_{\text{H}}=\frac{\left(\delta_{\text{C}}-\delta_{\text{H}}\right)}{2}\left\{\frac{{\left[\text{G}\right]}_{\text{t}}}{{\left(\text{H}\right)}_{\text{t}}}+1+\frac{1}{{\text{K}}_{\text{s}}{\left[\text{H}\right]}_{\text{t}}}\pm \sqrt{{\left(\frac{{\left[\text{G}\right]}_{\text{t}}}{{\left(\text{H}\right)}_{\text{t}}}+1+\frac{1}{{\text{K}}_{\text{s}}{\left[\text{H}\right]}_{\text{t}}}\right)}^{2}-4\frac{{\left[\text{G}\right]}_{\text{t}}}{{\left[\text{H}\right]}_{\text{t}}}}\right\}$$ (10)

Among them, H, G, C represent host, guest and complex, respectively. [H]_t_ and [G]_t_, denote total concentration of host molecule and guest molecule at initial stage. [H] denotes concentration of host at final stage. [G] denotes concentration of guest at final stage. [C] denotes concentration of complex at final stage. δ is the observed chemical shift. δ_H_ and δ_G_ are variation of chemical shift of host and guest, respectively. Ks is the binding constant in a balanced state. Eqs. 5–7 are derived from the Eq. 8. The δ-δ_H_ and δ_C_-δ_H_ were determined by the experiment. The Ks was calculated by the Eq. 10.

#### Nuclear Magnetic Resonance Spectroscopy

^1^H NMR and 2D ROESY NMR spectra were performed on a Bruker Advance III HD spectrometer (600 MHz, Bruker BioSpin, Switzerland) fitted with a 5-mm TCI probe at 298 K, and TMS used as the calibration signal. The solvent in the experiment was D_2_O (99%), CDCl_3_ (99%), or DMSO-d_6_ (99%).

#### Water Solubility Experiment

Disodium terephthalate could be used as an internal standard to measure the water solubility of the AD-CBD/BIO-CD inclusion complex. The D_2_O buffer of pH 7.2 was used to prepare the disodium terephthalate solution required for the experiment. The excessive AD-CBD/BIO-CD inclusion complex was added to the solution subsequently. The ^1^H NMR spectrum of AD-CBD/BIO-CD inclusion complex was obtained on NMR spectrometer after stirring for 1 h, and water solubility was calculated through integral ratio.

#### pH-dependent Drug Release Studies

Different release media (pH 1.2 HCl, pH 4.5 acetate buffer, pH 6.5, and 7.4 phosphate buffer) were applied to test the drug release curve of AD-CBD-loaded BIO-CD samples. Briefly, a total of 3.0 ml sample solution immersed in 100 ml of the buffer solutions by using an intelligent dissolution tester (Tianjin University Radio Manufacturing Factory, ZRS-8G, Tianjin, PR China) use basket method 100 rpm. UV-Vis analysis need to take out 3 ml of the solution within a predetermined time interval, while supplementing with fresh buffer. UV-Vis could determine the amount of AD-CBD released. Each experiment was carried out in triplicate.

#### Cytotoxicity Test

HeLa, HepG2, LO2, and A549 cell lines were obtained from Experimental Animal Center of Kunming University of Science and Technology (Kunming, China). Cell lines were cultured in DMEM medium at 37°C and humidified 5% CO2, which contained 10% fetal bovine serum (FBS), 100 U/ml penicillin and 100 μg/ml streptomycin. Cells were collected when they were in the exponential growth phase and used for cytotoxicity testing and cell uptake experiments. The counting chamber method was performed for cell counting. As detailed below.

MTT (3-(4,5-dimethylthiazole)-2-yl)-2,5-diphenyltetrazolium bromide) analysis was performed using HeLa, HepG2, A549, and LO2 cell lines to evaluate the cytotoxicity of BIO-CD, AD-CBD, and the inclusion complex of BIO-CD and AD-CBD *in vitro*. Firstly, the extracted cells were seeded into 96-well plate (1.0 × 10^4^ cells per well). Then, they were incubated for 24 h and treated with different concentrations of drug samples and positive controls for 48 h. The cell culture medium of each well was discarded, and 20 μl of MTT solution (5 mg ml^−1^ in PBS) was added, and continued to incubated for 4 h. A microplate reader was used to measure the absorbance at 490 nm. In this study, the experimental and control groups were carried out in triplicate, and the cell viability was represented by the absorbance relative to the untreated control groups. The cell survival rate was computed by the following Eq. 11:

$$\text{Cell Viability}\left(\%\right)=\frac{{\text{OD}}_{\text{exp}}-{\text{OD}}_{\text{blank}}}{{\text{OD}}_{\text{control}}-{\text{OD}}_{\text{bslank}}}\times 100$$ (11)

The absorbances of samples, blanks, and control experiments were represented by OD_exp_, OD_blank_, and OD_control_, respectively.

#### BIO-CD Targeting Verification Experiment

Different concentrations (0.001, 0.005, and 0.010 mM) of biotin were added into culture medium previously to verify the cancer targeting ability of biotin. The cells were cultured at 37°C with 5% CO_2_ for 24 h to design different levels of biotin to combine with the receptors on the surface of biotin cancer cells. Then new culture medium containing different concentrations of CBD, AD-CBD, BIO-CD, AD-CBD/BIO-CD were added to the cells and continued to cultivate for 48 h. Finally, we used the previous mentioned method to measure the OD value and calculate the IC_50_ value of CBD, AD-CBD, BIO-CD, and AD-CBD/BIO-CD.

#### Selective Cellular Uptake

Through cell uptake studies on Hela, A549, and LO2 cells, the selective targeting properties of BIO-CD were determined. Cells were inoculated into culture dishes (20 mm, PS, 5,000 cells/dish), and then incubated overnight. The BIO-CD loaded with Rhodamine B was incubated with cells for 6 h. As Rhodamine B yields red fluorescence, it could be used to examine the intracellular localization of BIO-CD particles. The nucleus was identified by Hoechst staining, which yields blue fluorescence. Confocal laser scanning microscopy (CLSM, Eclipse Ti, Nikon) was used to record the intracellular localization of the samples. All cells were carried out in triplicate.

#### Cellular Uptake Assays

Rhodamine B (20 μg ml^−1^), Rhodamine B/BIO-CD inclusion complex (or Rhodamine B/β-CD inclusion complex) (20 equivalents) were co-cultured with LO2, HepG2 and Hela cells at 37°C for 2 h. Subsequently, cells washed three times with PBS, and cell uptake efficiency was observed with an inverted fluorescence microscope.

## Result and Discussion

### Job Plot Analysis

**FIGURE 1:**
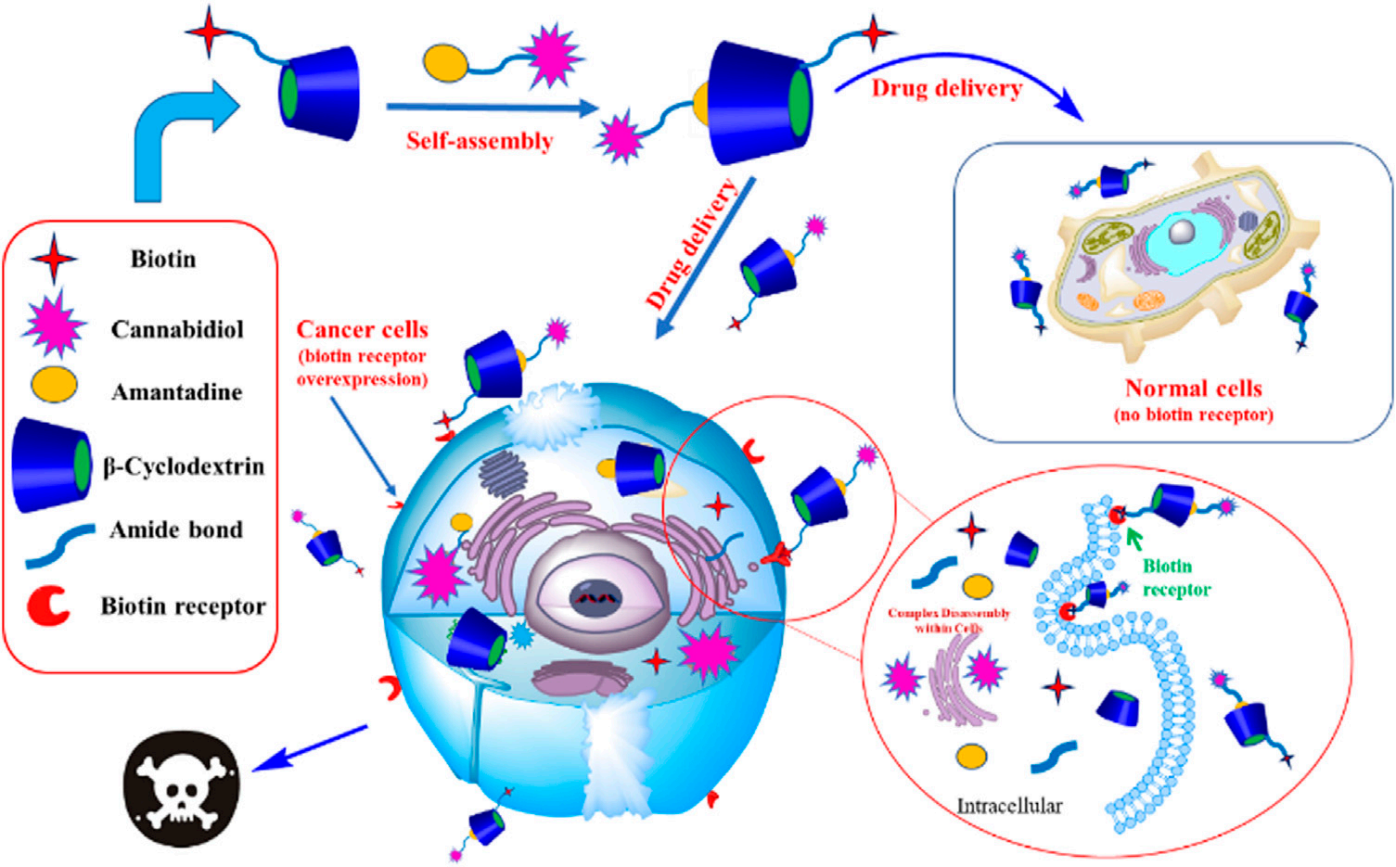
The preparation process of AD-CBD/BIO-CD complex and the mechanism of killing cancer cells.

**FIGURE 2:**
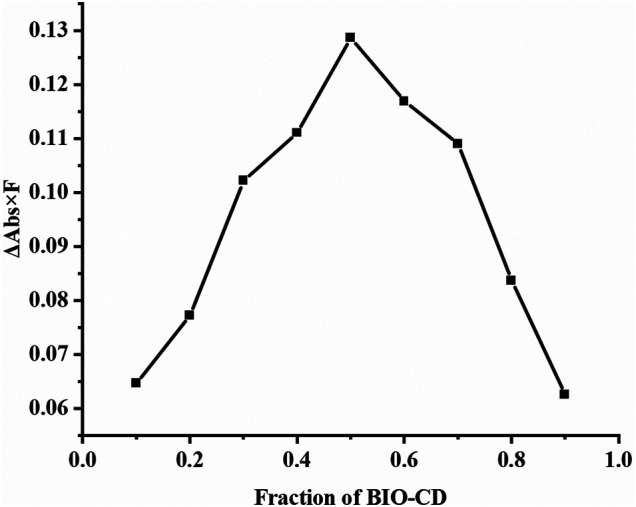
Job plot for the inclusion system of AD-CBD/BIO-CD at λ = 280 nm [(AD-CBD) + (BIO-CD) = 1.0 × 10^–3^ mol∙L^−1^] in methanol-water solutions (55: 45, v/v).

Job plot analysis can confirm the stoichiometric ratio of the inclusion complex. The Job plot of the AD-CBD/BIO-CD was shown in Figures 1, 2. Based on this method, the F value associated with the maximum value of ΔAbs × F was considered the stoichiometric proportion. The peak values of ordinate occurred at 0.5 as shown in Figure 2, which demonstrated that the stoichiometric ratio of the host and guest was 1:1. This was consistent with the results of the previous study that adamantane and ß-cyclodextrin could form a 1:1 inclusion complex.

### Characterization Results for AD-CBD

^1^H NMR spectra of amantadine-cannabidiol (AD-CBD) is shown in Supplementary Figure S2. ^1^H NMR (600 MHz, Chloroform-d): δ 6.56 (s, 1H), 6.45–6.34 (m, 1H), 6.03 (d, J = 6.0 Hz, 1H), 5.54 (m, 1H), 4.67–4.51 (m, 1H), 4.48–4.35 (m, 1H), 3.60–3.40 (m, 1H), 2.90 (s, 4H), 2.48 (m, 2H), 2.45–2.38 (m, 1H), 2.20 (m, 1H), 1.98 (d, J = 2.9 Hz, 5H), 1.87–1.70 (m, 5H), 1.67 (s, 3H), 1.63 (m, 3H), 1.56 (m, 2H), 1.32–1.23 (m, 4H), 0.87 (t, J = 6.0 Hz, 3H). ^13^C NMR (151 MHz, Chloroform-d): δ 171.84, 170.25, 162.65, 155.64, 149.58, 147.17, 124.43, 123.39, 114.68, 111.51, 110.87, 45.79, 36.59, 36.30, 35.57, 35.00, 31.88, 31.59, 31.23, 30.73, 30.52, 30.37, 29.67, 29.48, 29.37, 28.36, 27.90, 23.73, 23.65, 22.57, 22.47, 22.38, 19.96, 19.61, 14.05. (Supplementary Figure S3) ESI-MS: m/z = 547.3669 [M-H]^-^ (Supplementary Figure S4).

### Scanning Electron Microscope Analysis

**FIGURE 3:**
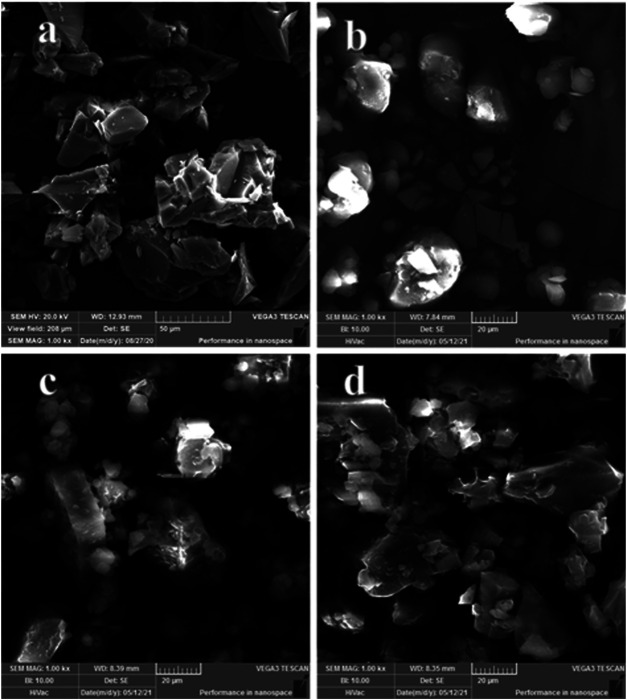
SEM microphotographs: **(A)** BIO-CD; **(B)** AD-CBD; **(C)** the physical mixture (AD-CBD: BIO-CD = 1:1), and **(D)** AD-CBD/BIO-CD inclusion complex.

Microscopic morphological changes of AD-CBD before and after binding with BIO-CD were shown by SEM analysis (Figure 3). The irregular particles in Figure 3A were BIO-CD, and the irregular crystal particles in Figure 3B were AD-CBD. The AD-CBD and BIO-CD particles were clearly observed in Figure 3C. Conversely, Figure 3D indicated that after the solid inclusion complex was formed, the morphological features of AD-CBD were disappeared completely to exist in a significant broken rock-shaped particles. Thus, the morphological features of AD-CBD/BIO-CD inclusion complex were significantly different from those of their physical mixtures.

### Fourier Transform Infrared Analysis

**FIGURE 4:**
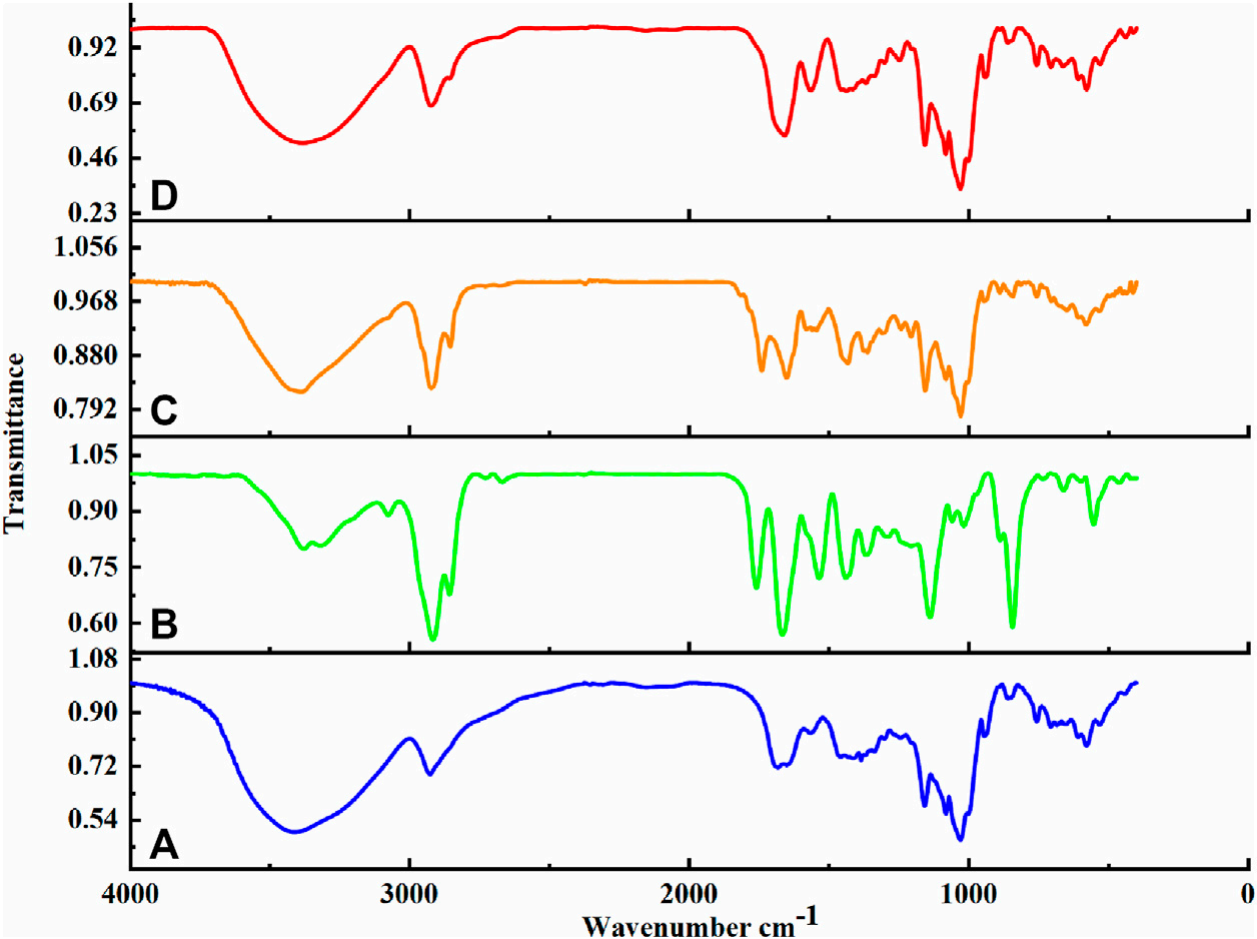
FT-IR spectrum of **(A)** BIO-CD; **(B)** AD-CBD; **(C)** the physical mixture (AD-CBD: BIO-CD = 1:1); **(D)** AD-CBD/BIO-CD inclusion complex.

The changes in the position, frequency and shape of the characteristic peaks of many groups in the molecule can be identified by FT-IR method. Consequently, it is extensively applied in the research of host-guest interaction. The FT-IR results were shown in Figure 4 (BIO-CD (Figure 4A), γs_(O-H)_ = 3,410 cm^−1^, γs_(C-H)_ = 2,925 cm^−1^, δs_(H-O-H)_ = 1,670 cm^−1^, γs_(C-O)_ = 1,156 cm^−1^, γs_(C-O-H)_ = 1,033 cm^−1^; AD-CBD (Figure 4B), γs_(O-H)_ = 3,367, 3,321 cm^−1^, γs_(C-H of benzene ring)_ = 3,074 cm^−1^, γs_(C-H)_ = 2,918, 2,850 cm^−1^, γs_(C-C of benzene ring)_ = 1,656 cm^−1^, 1,539 cm^−1^, 1,429 cm^−1^, δs_(C-H)_ = 1,350 cm^−1^, γs_(C-O)_ = 1,132 cm^−1^).

In Figure 4C, sharp characteristic peaks of AD-CBD were shown clearly in the AD-CBD and BIO-CD physical mixture. The infrared spectra of AD-CBD/BIO-CD and BIO-CD were similar, which was worthy of our attention. As shown in Figure 4D, the peaks of AD at 3,100–2,850 and 1,132 cm^−1^ were all almost disappeared. The results indicated that AD-CBD entered the cavity of BIO-CD completely or partially, which limited the vibration of the corresponding functional groups. These results further certificated the successful formation of AD-CBD/BIO-CD inclusion complex.

### Powder X-Ray Diffraction Analysis

**FIGURE 5:**
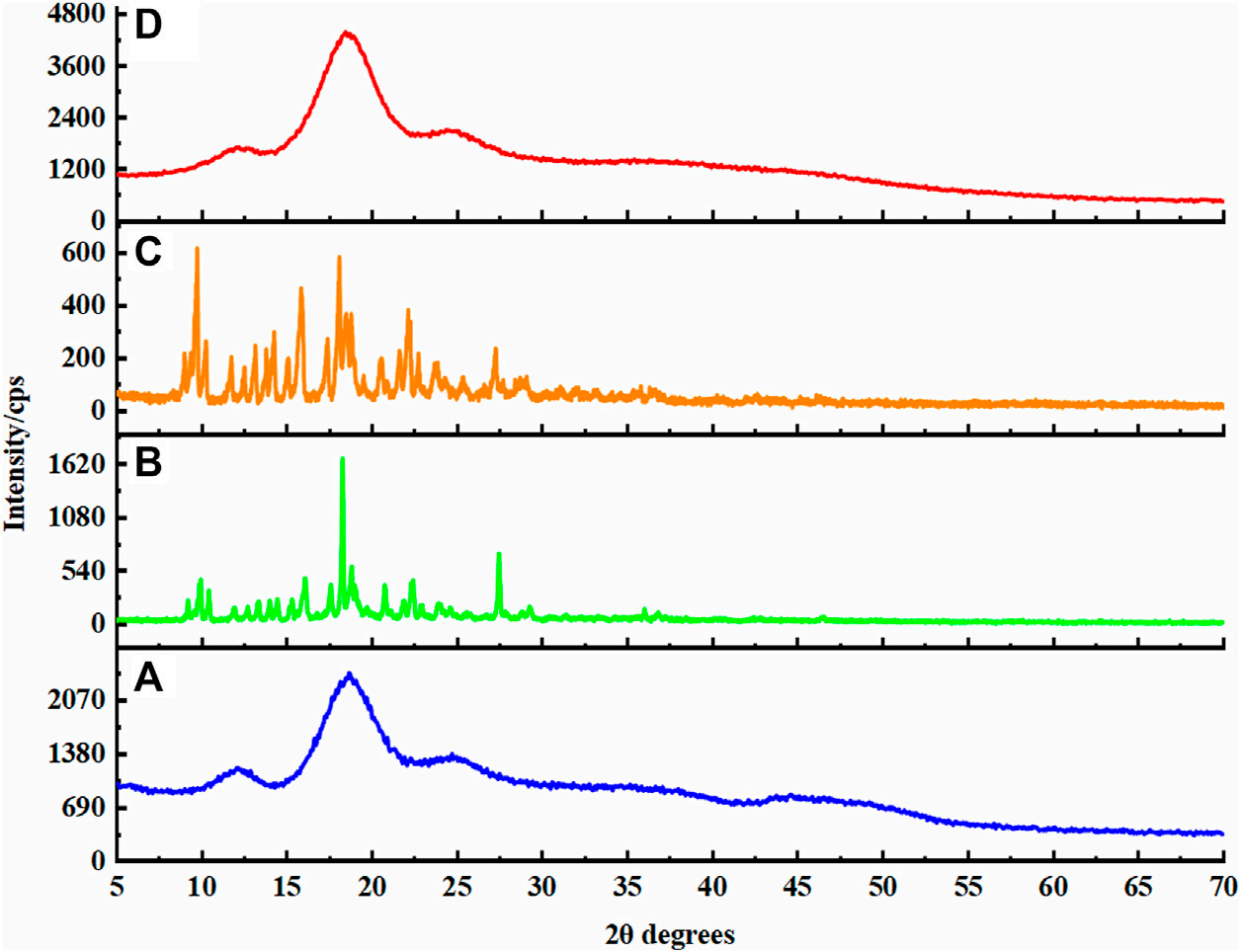
XRD patterns: **(A)** BIO-CD; **(B)** AD-CBD; **(C)** the physical mixture (AD-CBD: BIO-CD = 1:1); **(D)** AD-CBD/BIO-CD inclusion complex.

The crystal form information of AD-CBD/BIO-CD inclusion complex could be obtained by powder X-ray diffraction analysis. As shown in Figure 5, the XRD pattern of BIO-CD exhibited amorphous pattern (Figure 5A), while AD-CBD displayed sharp absorption peak at 2θ = 9.7, 10.1, 13.1, 14.9, 18.8, 22.1, and 33.2°, indicating its crystal structure (Figure 5B). The XRD pattern of AD-CBD and BIO-CD physical mixture (1:1) kept similar characteristic crystal peaks, but the intensity was far below than AD-CBD (Figure 5C). Nevertheless, there was no characteristic peak of AD-CBD, or BIO-CD was observed in the pattern of the inclusion complex, which indicated that the AD-CBD/BIO-CD inclusion complex was formed (Figure 5D).

### Thermal Analysis

**FIGURE 6:**
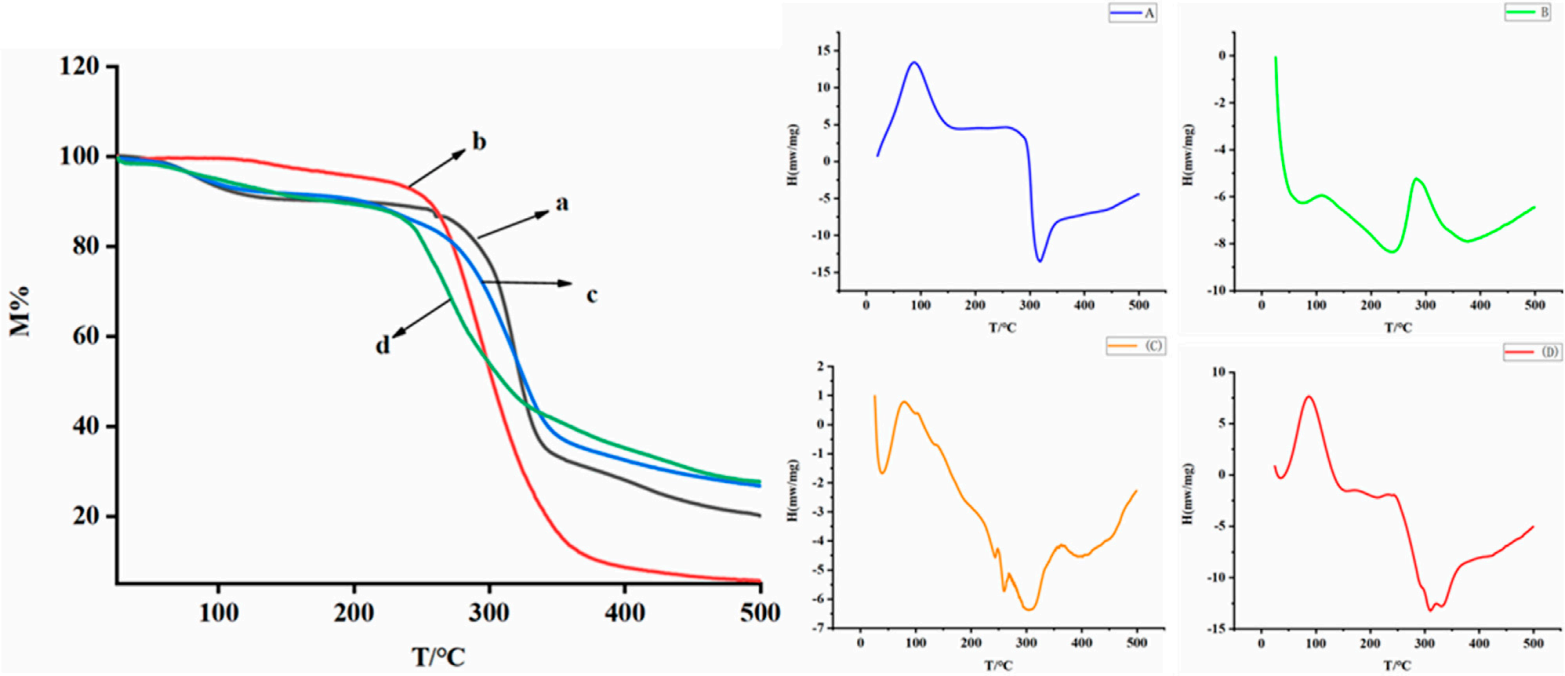
TG curves of **(A)** BIO-CD, **(B)** AD-CBD, **(C)** the physical mixture (AD-CBD: BIO-CD = 1:1); **(D)** AD-CBD/BIO-CD inclusion complex and DSC thermograms of **(A)** BIO-CD, **(B)** CBD, **(C)** their 1:1 physical mixture and **(D)** inclusion complex.

Common methods for studying the physical states of inclusion complex include TG and DSC analysis. The thermal properties of the AD-CBD, BID-CD, and their solid inclusion complex and physical mixtures were obtained by TG and DSC analysis. TG curves were shown in Figure 6, the host molecule BIO-CD decomposed rapidly and then the decomposition rate slowed down from 255°C to 360°C (Figure 6A). AD-CBD began to disintegrate at about 235°C (Figure 6B). The quality physical mixture began to reduce at about 250°C (Figure 6C). Nevertheless, the thermal decomposition points of the solid inclusion complex appeared at around 210°C and started to decompose slowly (Figure 6D). These results further indicated that the inclusion complex had been formed successfully.

DSC analysis revealed the thermal properties of the inclusion complex between AD-CB and BIO-CD. As shown in Figures 6A,B BIO-CD had a clear endothermic peak at 86°C, an exothermic hollow at 316°C (Figure 6A), and AD-CBD showed a sharp endothermic peak at 282°C (Figure 6B). As shown in Figure 6C, the physical mixture of 1:1 was almost exhibited a combination of features of the two natural components, which occurred endothermic peaks at 77°C and 268°C, and gave exothermic hollows at 303°C. In Figure 6D, the characteristic DSC behavior of their inclusion complex was very similar to that of BIO-CD, where the representative AD-CBD endothermic peak was completely disappeared at 282°C. Which suggested the formation of the inclusion complex strongly.

### ^1^H Nuclear Magnetic Resonance Titration Assays

**FIGURE 7:**
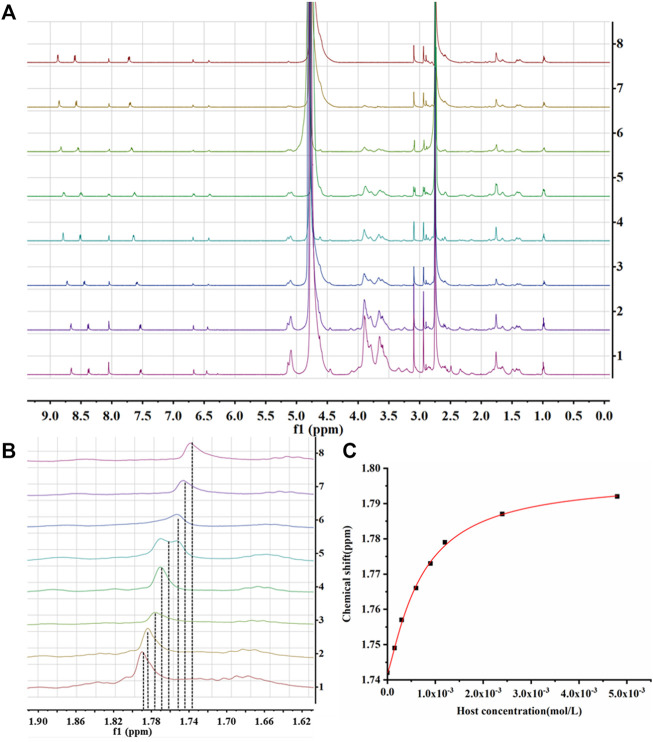
**(A)** ^1^H NMR spectra in D_2_O at 25°C (From top to bottom, host: guest was 0–1.6); **(B)** The enlarged view of 1.62 ∼ 1.90 ppm of **(A)**; **(C)** The titration curve of AD-CBD/BIO-CD inclusion complex.

^1^H NMR titration assays was used to measure the Ks values of inclusion complex. Samples with different concentration gradients (the host-guest ratio ranged from 0 to 1.6) were prepared, and their ^1^H NMR spectra were recorded. When the host molecule was present in the system, the inducing chemical shift of the AD-CBD protons were displayed in each spectrum (Figures 7A,B), which indicated the formation of inclusion complex in solution. The titration curve was drawn according to the chemical shift change of AD (about 1.80 ppm) in AD-CBD (Figure 7C). The titration curve was fitted on the basis of several chemical shift values, concentrations of host, and previous NMR binding models, and then calculated the Ks value of host-guest complex. The data was fitted to the NMR binding model, and the K_S_ value of the inclusion complex of AD-CBD/BIO-CD was determined as (4.8 ± 0.9) × 10^3^ M^−1^.

### Inclusion Modes of AD-CBD/BIO-CD

**FIGURE 8:**
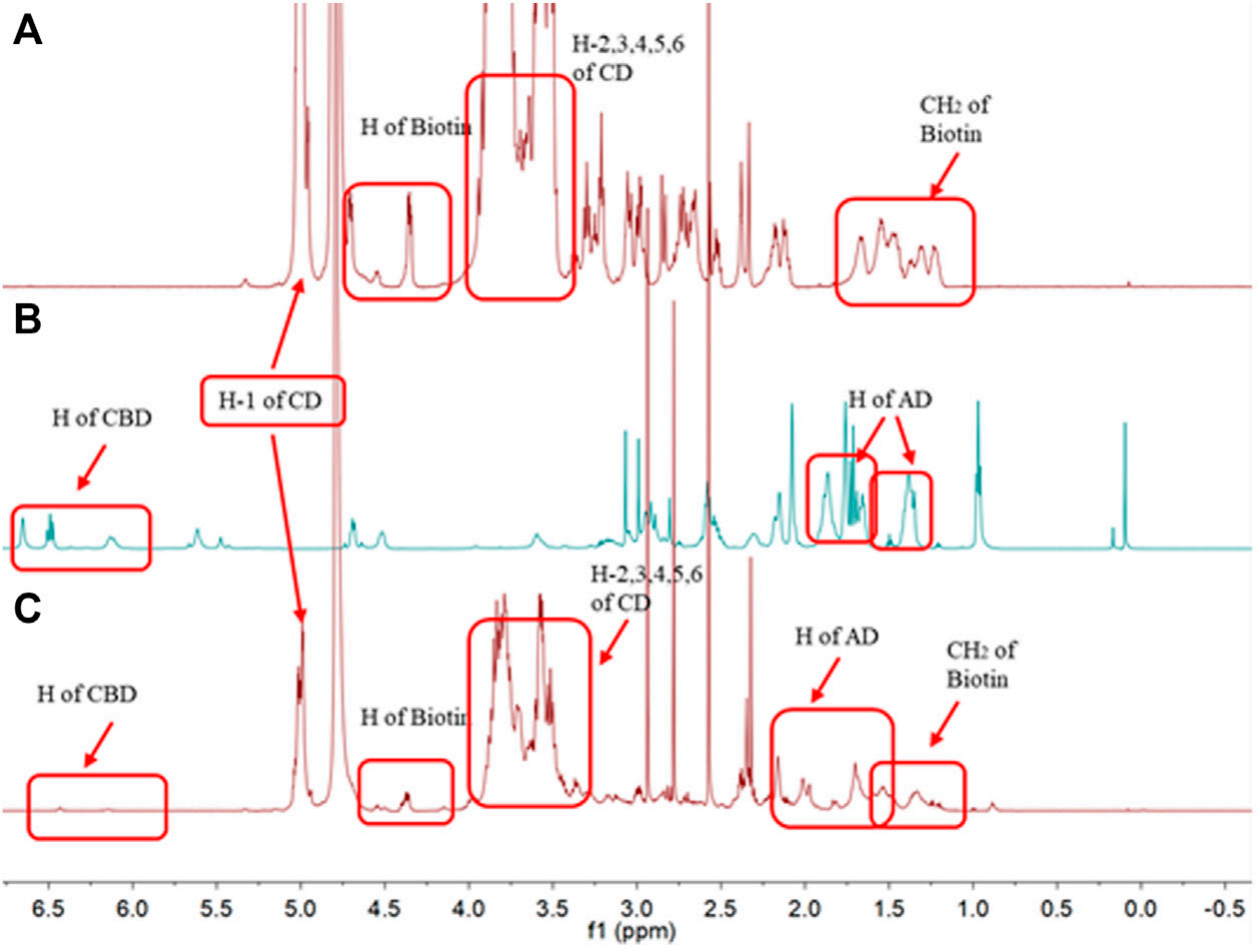
^1^H NMR spectra for **(A)** BIO-CD in D_2_O, **(B)** AD-CBD in CDCl_3_, and **(C)** the inclusion complex of AD-CBD/BIO-CD in D_2_O.

NMR is usually used for the analysis of inclusion modes of inclusion complex. Due to the poor water solubility of AD-CBD, the AD-CBD proton peaks could not observe (Figure 8) when D_2_O was used as a solvent in most cases. However, the ^1^H NMR evaluation of the AD-CBD/BIO-CD inclusion complex, characteristic absorption peak of the AD-CBD molecular clearly existed in D_2_O, which demonstrated that the inclusion complex of AD-CBD with BIO-CD was formed. Also, we observed that the solubility of AD-CBD/BIO-CD increased significantly than natural AD-CBD. We determined the chemical shift changes for protons of BIO-CD in the presence and absence of AD-CBD. The downfield displacement variation of BIO-CD H-1 to H-6 protons were summarized in Tables 1, 2, which ranging from 0.01 to 0.05 ppm.

**TABLE 1:** The chemical shift changes of BIO-CD protons formed inclusion complex with AD-CBD in D_2_O at 25°C.

Protons	Chemical shift (ppm)		
	δ _BIO-CD_	δ _complex_	Δδ (δ _complex_−Δ_BIO-CD_)
H-1 of BIO-CD	5.02	5.01	−0.01
H-2 of BIO-CD	3.62	3.58	−0.04
H-3 of BIO-CD	3.89	3.85	−0.04
H-4 of BIO-CD	3.55	3.52	−0.03
H-5 of BIO-CD	3.80	3.75	−0.05
H-6 of BIO-CD	3.75	3.72	−0.03

**TABLE 2:** The water solubility of AD-CBD and its inclusion complex in buffer solution (KH_2_PO_4_/K_2_HPO_4_, pH 7.0, 25°C).

Inclusion complex	Water solubility of CBD (mg·ml^−1^)	Fold increase than native CBD
CBD	6.27 × 10^–5^	1
AD-CBD	6.50 × 10^–5^	1.04
BIO-CD/AD-CBD	3.16	5.04 × 10^4^
β-CD/CBD	2.10	3.35 × 10^4^
HP-β-CD/CBD	4.90	7.81 × 10^4^

**FIGURE 9:**
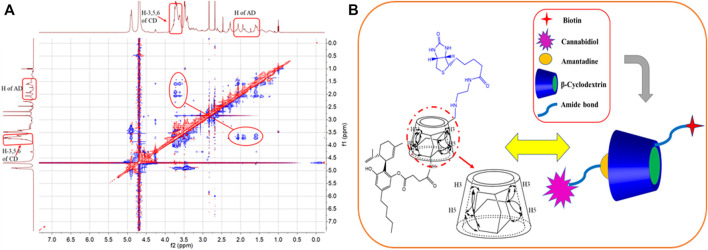
**(A)** The 2D ROESY spectrum for the inclusion complex of AD-CBD and BIO-CD in D_2_O. **(B)** Possible inclusion mode between AD-CBD and BIO-CD.

The study of inter and intra-molecular interaction are usually employed by the method of 2D NOESY or ROESY NMR spectra. When the contact space of the two protons is within 0.5 nm, NOE cross peaks could be observed between of them. 2D ROESY NMR spectrum in D_2_O was recorded for explaining the inclusion mode of AD-CBD/BIO-CD inclusion complex. It was shown that the protons of AD and the H-3, 5 protons of ß-CD were correlated in the 2D ROESY spectrum of AD-CBD/BIO-CD complex (Figure 9A). Experimental results demonstrated that the AD of AD-CBD was included in the cavity of BIO-CD. We inferred the possible inclusion patterns of AD-CBD with BIO-CD based on these analysis results as shown in Figure 9B.

### Water Solubility

For the water solubility measurement of the AD-CBD/BIO-CD inclusion complex, disodium terephthalate can be used as an internal standard. The prep, disodium terephthalate can be used as an internal standard. The preparation of a certain concentration (10 mM) disodium terephthalate solution can be carried out in deuterated buffer solution at pH 7.2. Added excess AD-CBD to the solution and stirred for 1 h. Subsequently, the NMR integral ratio was carried out to evaluate the water solubility of the AD-CBD/BIO-CD inclusion complex, and the water solubility was obtained to be 3.16 mg ml^−1^. Compared with natural CBD (ca. 6.27 × 10^–5^ mg ml^−1^), the water solubility was increased about 5.04 × 10^4^ folds.

### *In Vitro* Release Property

**FIGURE 10:**
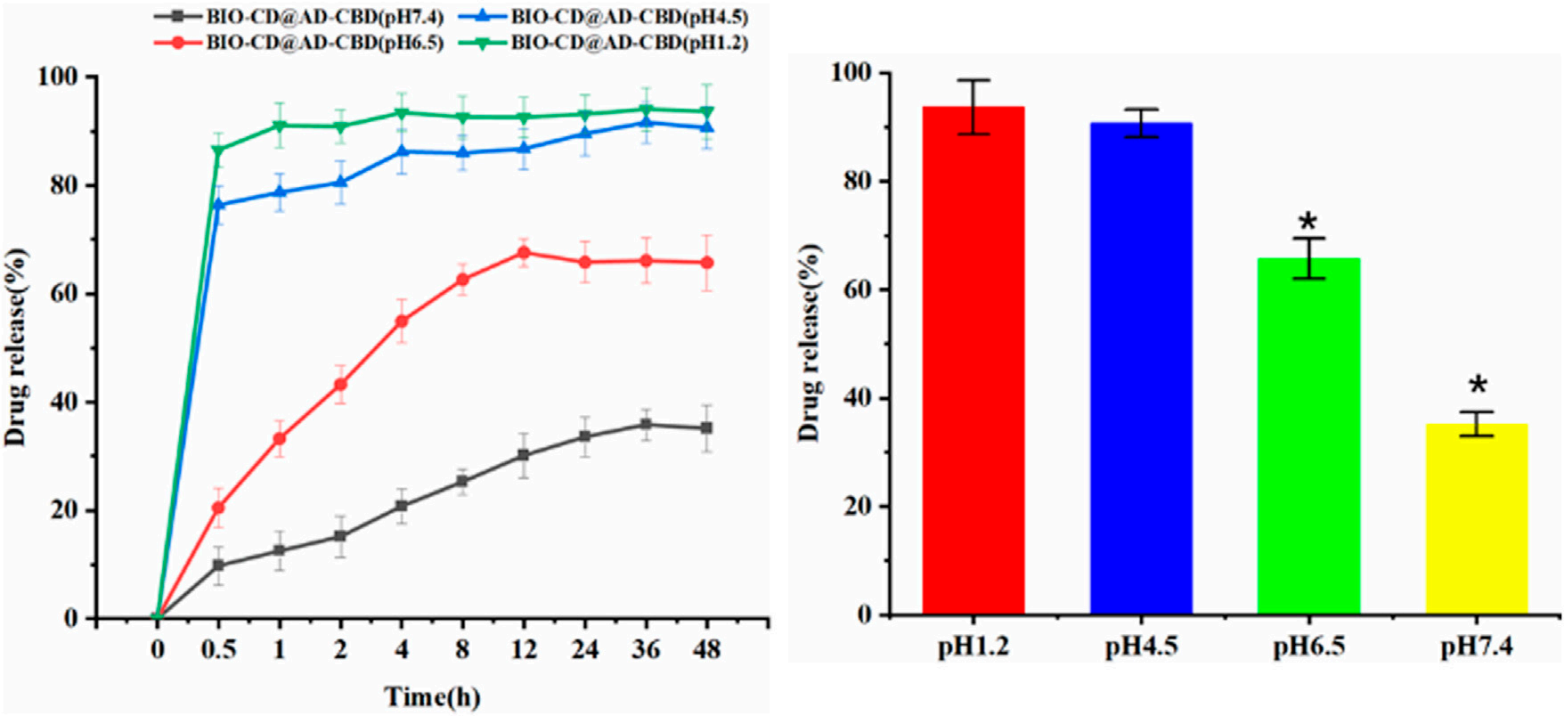
Release profiles of guest molecular under different pH buffer solutions.

We tested the effect of pH on drug release, was aimed to observe whether BIO-CD has the targeted ability to deliver chemotherapeutics to cancerous tissues. In order to explore the drug release laws at different pH values (pH 7.4, 6.5, 4.5, and 1.2), four buffer solutions were used to simulate the micro environment of normal human tissues, tumor tissues, organelles and gastric juice at 37°C. After 1 h, we observed that the drug release was enhanced at the acidic pH (pH 4.5 acetate buffer and 1.2 HCl) (Figure 10). In addition, the drug release under pH 6.5 and 7.4 phosphate buffers were further tested. We observed AD-CBD was released suddenly at acidic pH, while CBD was released lower at pH 7.4. Although the guest was relatively stable in the bloodstream (∼pH 7.4), it rapidly released in the weakly acidic micro environment of tumor, which indicated that this pH-mediated drug release mode was important for the designation of controlled drug delivery system. In addition, we recorded the stability of AD-CBD/BIO-CD in different pH media by UV-vis spectra. Supplementary Figure S5 UV-vis spectra indicated that there was no wavelength shift in pH 7.4 and 6.5, but shift in pH 4.5 and 1.2. Therefore, we could conclude that AD-CBD/BIO-CD can remain stable relatively in human plasma (∼pH 7.4) and cancer tissue (∼pH 6.5).

### *In Vitro* Cytotoxicity Studies

The MTT assay was used to evaluate the cytotoxicity of AD-CBD/BIO-CD inclusion complex against human cancer cell lines HepG2, Hela, and A549 and the human liver cell line LO2, respectively. Cisplatin was used as the positive drug. The calculation of IC_50_ value was shown in Table 3. The cytotoxicity of the inclusion compound was slightly lower than that of CBD, AD-CBD, and cisplatin *in vitro*. This may be due to the targeting effect of biotin on cancer cells that overexpressed biotin receptors. Compared with cisplatin, CBD, and AD-CBD, the selectivity of the inclusion complex was also reflected in the toxicity to tumors that significantly higher than the above three, while no toxicity to normal cells (the LO2 surface has no biotin receptor, IC_50_ value > 100), that confirmed its biosafety for a promising treatment. The above results could provide meaningful clues for the development of new formulations of CBD for clinical anticancer drug treatment. Although the anticancer activity *in vivo* and the distinct action mechanism of AD-CBD/BIO-CD inclusion complex were not revealed.

**TABLE 3:** IC_50_ (μM) of solid inclusion complex of AD-CBD/BIO-CD by MTT assay.

Entry	Samples	IC_50_ (μM)			
		HepG2	Hela	A549	LO2
1	CBD	15.71	14.14	9.81	29.21
2	AD-CBD	16.24	14.95	8.90	18.85
3	BIO-CD	>100	>100	>100	>100
4	AD-CBD/BIO-CD	12.72	13.26	4.59	>100
5	Cisplatin	13.58	13.70	15.34	22.77

### Verification Experiment of Cancer-Targeting of Biotin

**FIGURE 11:**
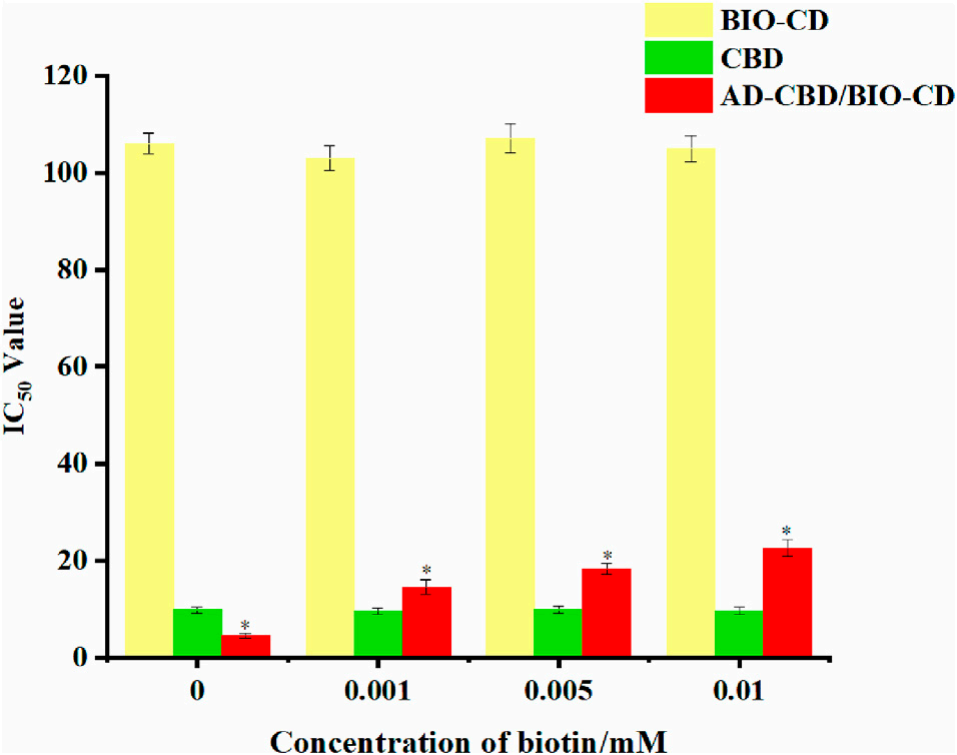
IC_50_ values of AD-CBD, BIO-CD, and AD-CBD/BIO-CD towards A549 cell lines under different concentrations of biotin (*p < 0.05).

In our study, we evaluated the IC_50_ values of the AD-CBD/BIO-CD inclusion complex on A549 which cultured with multiple concentrations of biotins. The IC_50_ values without biotin pretreatment were also tested. As shown in Figure 11, the IC_50_ value of the AD-CBD/BIO-CD inclusion complex gradually increased with the increase of the amount of biotin. It might be attributed to the gradual occupation of biotin receptors on the surface of cancer that resulted in a decrease of targeting ability of the inclusion complex. In addition, without targeting ability, IC_50_ values were almost unchanged for CBD, AD-CBD, and BIO-CD.

### Targeted Drug Delivery to Tumor Cell Models

The prerequisite for drug delivery is biocompatibility. In the cytotoxic test under different incubation times, the BIO-CD exhibited high biocompatibility (Table 3). Besides the established biodegradability of previous study, it also had biocompatibility of practical drug delivery applications. Since CBD enhanced cellular uptake by the biotin receptor mediated cellular uptake, the cytotoxicity of AD-CBD/BIO-CD was enhanced, as shown in the following experiments.

**FIGURE 12:**
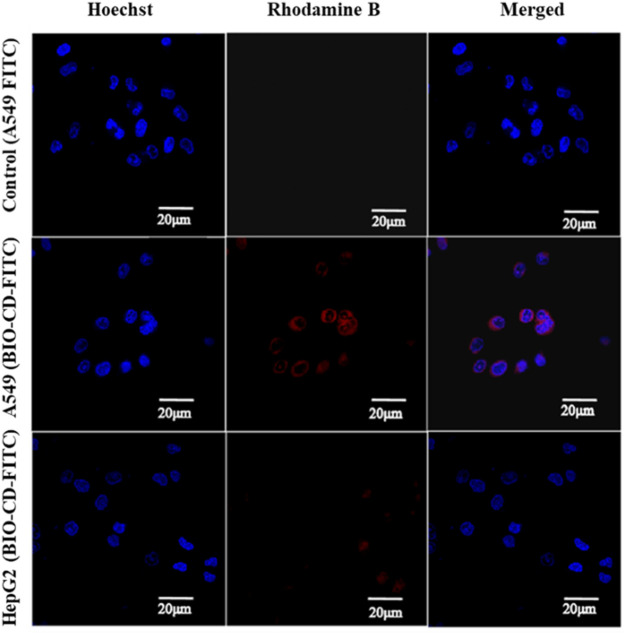
Targeted delivery of a model drug Rhodamine B using BIO-CD in HeLa cells and A549 cell models, showing the selective binding capability of BIO-CD to cells with biotin receptors.

Therefore, the feasibility of targeted drug delivery of using BIO-CD was evaluated in our study. The model drug Rhodamine B was used to demonstrate that the BIO-CD polymer delivered the drug selectively to the biotin receptor overexpressed cancer cells. The two cell lines of HeLa cervical cancer and A549 human lung cancer have different biotin receptor expression levels on the cell membranes, thence, they are used as cell models. From the confocal image, the copolymer loaded with Rhodamine B was combined with HeLa cells selectively. But the fluorescence of the cell surface of A549 was negligible, indicating the biotin receptor targeting ability of BIO-CD (Figure 12).

**FIGURE 13:**
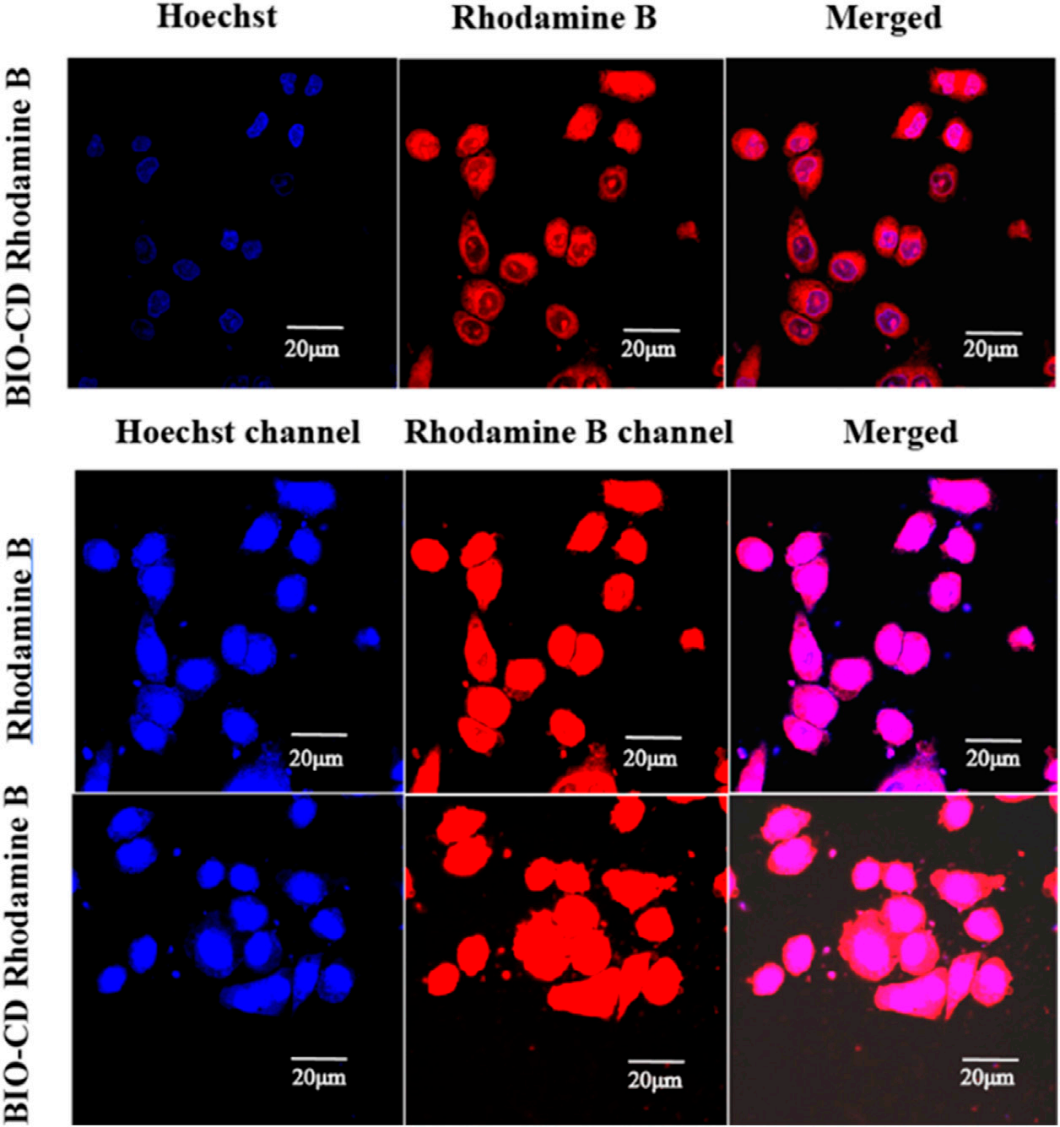
The CLSM images of A549 cells after 4 h of incubation with Rhodamine B-loaded BIO-CD. The cell nuclei are stained with Hoechst (blue fluorescence). Rhodamine B (red fluorescence) is visible.

Two model drugs Rhodamine B and Hoechst dyes were used to explain the selective drug delivery efficacy of BIO-CD. The microscopic images showed that Hoechst crossed the cell membrane, indicating that Hoechst entered the cytoplasm. We observed that massive Rhodamine B accumulated in the cell surface when incubated with HeLa cells for 4 h in the delivery experiment of Rhodamine B. This result was significantly higher than Rhodamine B without using BIO-CD (Figure 13). Consequently, it proved that the BIO-CD could bind to biotin receptors selectively, mediated the absorption and accumulation of Rhodamine B in cells. Due to the enhanced cellular intake of the polymer-drug complex, the inclusion complex of AD-CBD/BIO-CD has much higher cytotoxicity than free AD-CBD.

### Cellular Uptake Assays

**FIGURE 14:**
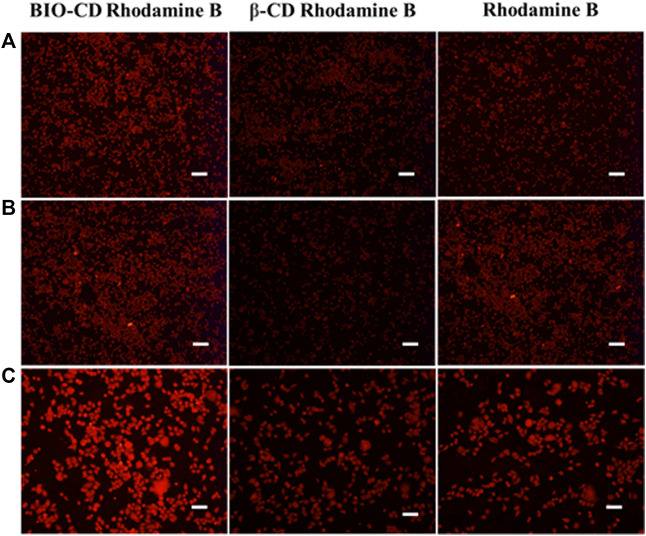
Targeting cellular delivery of Rhodamine B by BIO-CD. Fluorescence imaging for cellular uptake of BIO-CD load Rhodamine B in HepG2, Hela and LO2 cells. **(A)** LO2, **(B)** HepG2, **(C)** A549. The concentrations of free Rhodamine B (20 μg ml^−1^) and inclusion complex mentioned are as per mole of Rhodamine B. Scale bars are 25 μm.

We studied the capability of BIO-CD to promote the uptake of guest molecules in tumor cells. Rhodamine B was used as a model drug. Hela, HepG2, and LO2 cells were incubated with the inclusion complex of BIO-CD or ß-CD in media for 1 h. Meanwhile, we had observed a similar situation with ß-CD in normal cells LO2. As shown in Figure 14, after Rhodamine B entered the cavity of BIO-CD, it could enhance the efficiency of cell fluorescence imaging, while ß-CD was opposite. This might attribute to that BIO-CD specifically binded to the over-expressed biotin receptor on the surface of tumor cells, which resulted in the enhanced drug uptake. Because ß-CD did not have the targeting ability of biotin, and LO2 cell surface did not have the over-expressed biotin receptor, these possibly reduced the uptake capacity of cells. Data mentioned above confirmed BIO-CD successfully encapsulated guest drug and specifically bound to the biotin receptor to enhance the cellular uptake.

## Conclusion

In the pharmaceutical and biomedical materials industry, the supramolecular drug carrier with targeted function has a very promising research value. A host-guest complex of BIO-CD/AD-CBD based on supramolecular self-assembly, with targeted capability and pH-dependent release was designed and prepared in this study. Among them, inclusion complexes of AD-CBD/BIO-CD exhibits excellent anti-cancer activity, biocompatibility, controlled release ability, and selective delivery ability to the cancer cells, overexpressing biotin receptors. Therefore, this targeted host-guest complex is a promising approach for the treatment of cancer.

## Data Availability

The raw data supporting the conclusions of this article will be made available by the authors, without undue reservation.
