# Supplementary material for: Self-Assembly System Based on Cyclodextrin for Targeted Delivery of Cannabidiol
Source: Front Chem. 2021 Nov 8;9:754832. doi: 10.3389/fchem.2021.754832 (PMC8606678; doi:10.3389/fchem.2021.754832)
Supplement: Supplementary file 1 [file Presentation1.pdf]

Self-assembly system based on cyclodextrin for targeted delivery of cannabidiol

Panyong Zhu[1,a], Pin Lv [1,a, b,], Yazhou Zhang [a], Rongqiang Liao [c,]*, Jing Liu [d], Rong Guo [b], Xuan Chen [b], Xiali Liao [a], Chuanzhu Gao [a], Kun Zhang [e], Ming Yang [b,]*, Bo Yang [a,]**

a: Faculty of Life Science and Technology, Kunming University of Science and Technology, Kunming 650500, PR China.
b: Industrial Crop Research Institute, Yunnan Academy of Agricultural Sciences, Kunming, 650205, PR China.
c: Department of pharmacy, Chongqing Emergency Medical Center, Chongqing University Central Hospital, Chongqing 400014, PR China.
d: The Affiliated of Stomatology, Kunming Medical University, Kunming 650000, PR China.
e: School of Agriculture, Yunnan University, Yunnan University, Kunming 650500, PR China.

Contents

1. Standard curve of AD-CBD 2
2. ^{1}H NMR spectra of AD-CBD 2
3. ^{13}C NMR spectra of AD-CBD 3
4. ESI-MS of AD-CBD 3
5. The stability of AD-CBD/BIO-CD in different pH media 4

∗ Corresponding author. E-mail addresses: lvpin_342526463@qq.com (P. Lv), Rongqiang Liao liaorongqiang@126.com(R. Liao), yangbo6910@sina.com (B. Yang), ymhemp@163.com (M. Yang).
1. These authors have contributed equally to this work and share first authorship.

Experimental section.

1. Standard curve of AD-CBD

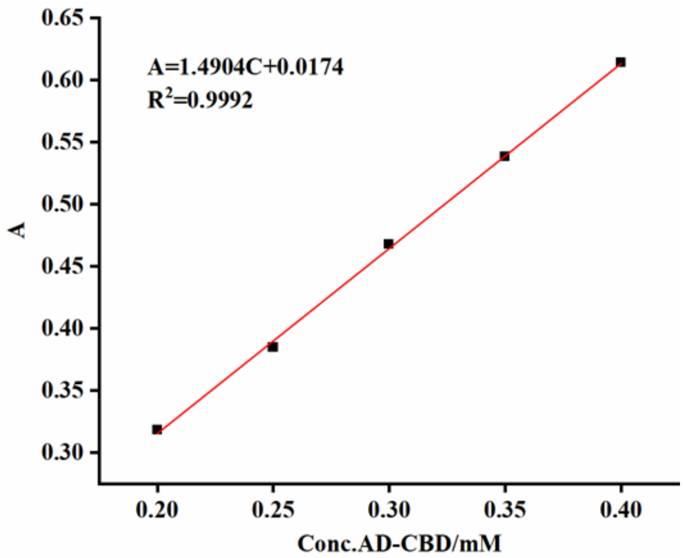

Fig. S1. Standard curve of AD-CBD.

2. ^{1}H NMR spectra of AD-CBD.

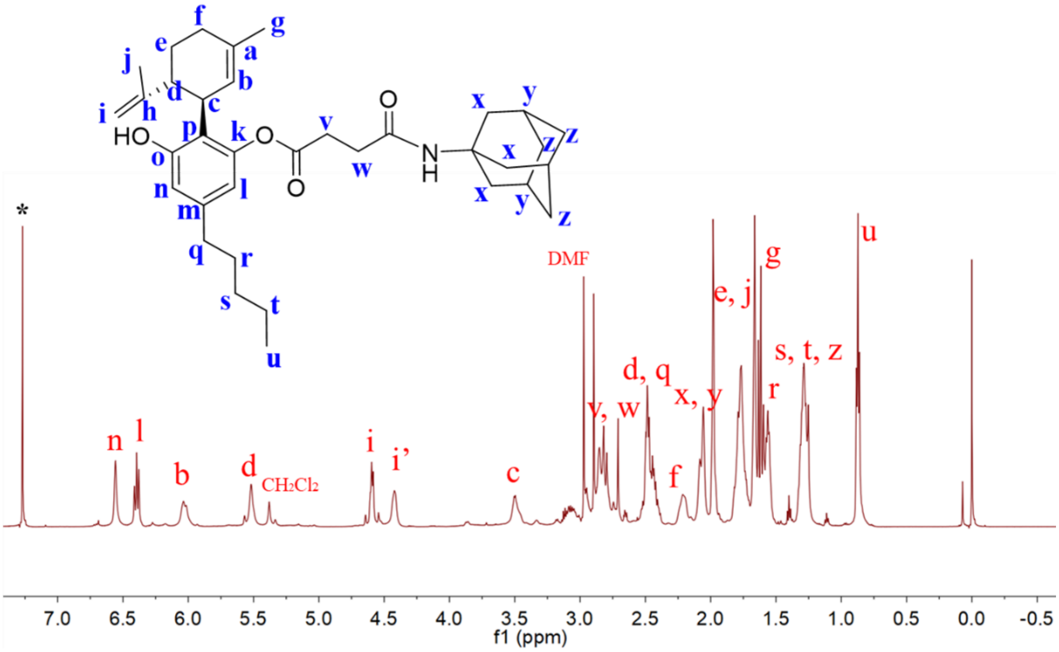

Fig. S2. ^{1}H NMR spectra of AD-CBD.

3. ^{13}C NMR spectra of AD-CBD

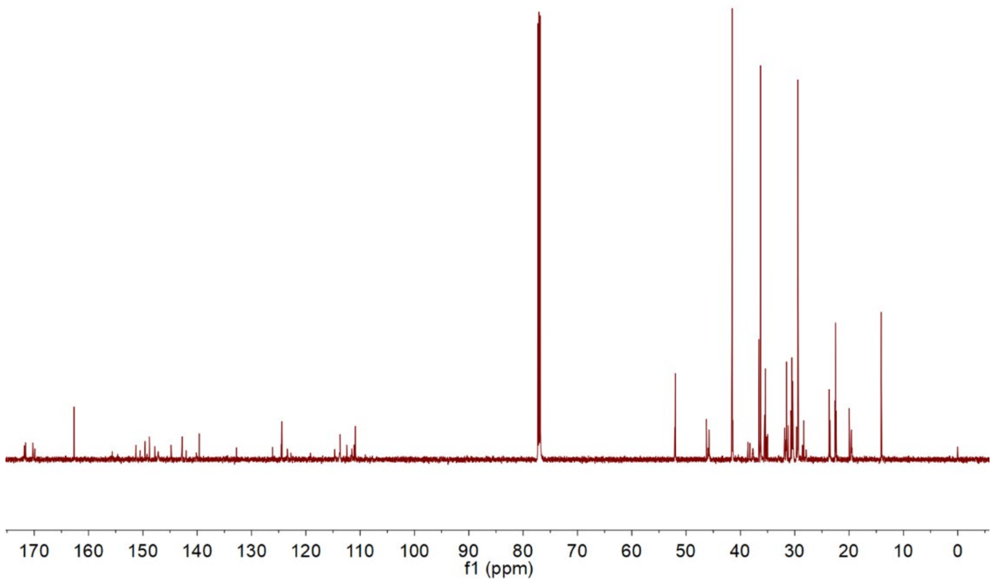

Fig. S3. ^{13}C NMR spectra of AD-CBD.

4. ESI-MS of AD-CBD

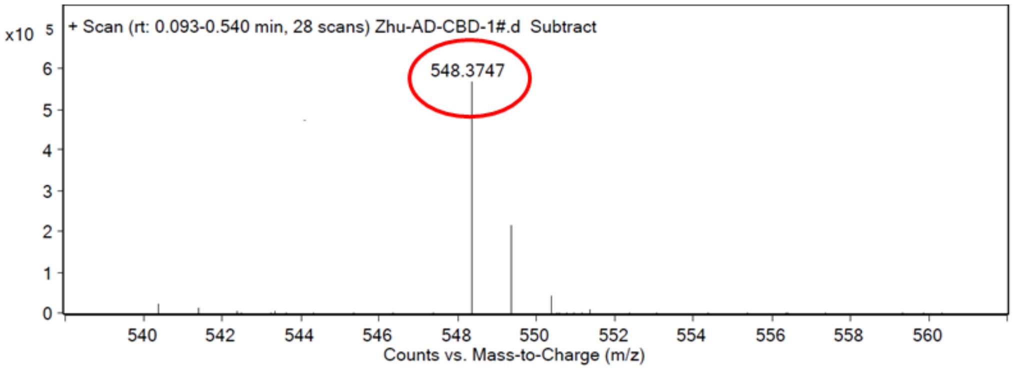

Fig. S4. ESI-MS of AD-CBD.

5. The stability of AD-CBD/BIO-CD in different pH media

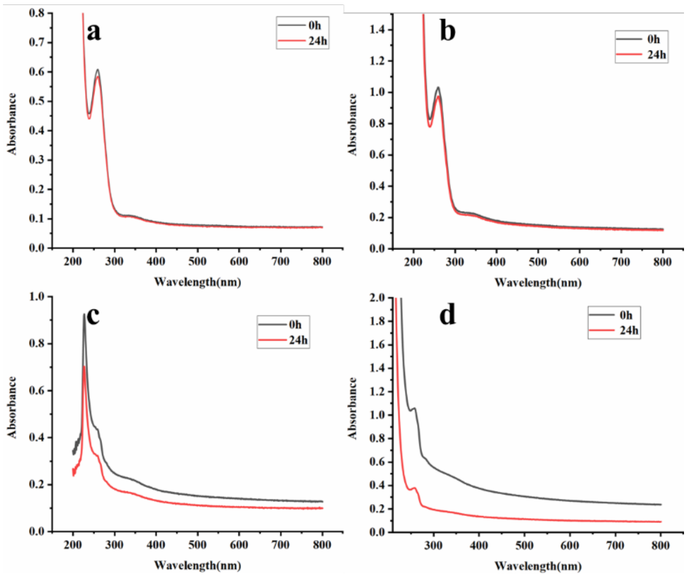

Fig. S5. The stability of AD-CBD/BIO-CD in different pH media
